# Progress and challenges in implementing HIV care and treatment policies in Latin America following the treatment 2.0 initiative

**DOI:** 10.1186/s12889-015-2565-9

**Published:** 2015-12-19

**Authors:** Freddy Perez, Bertha Gomez, Giovanni Ravasi, Massimo Ghidinelli

**Affiliations:** HIV, Hepatitis, Tuberculosis and Sexually Transmitted Infections Unit; Communicable Diseases and Health Analysis Department, Pan American Health Organization, 525 23rd Street NW, Washington, DC 20037-2895 USA

**Keywords:** Treatment 2.0 initiative, Latin America, Comprehensive response to HIV/AIDS, Joint Review Missions

## Abstract

**Background:**

The Pan American Health Organization provides technical cooperation to countries in Latin America and the Caribbean for the scale-up of HIV care and treatment based on the Treatment 2.0 initiative. Fourteen Joint Review Missions (JRMs) were conducted between March 2012 and October 2014. Evaluating the degree of implementation of the recommendations of the JRMs and their impact on health policies, would help countries identify their gaps and areas for priority interventions.

**Methods:**

A descriptive analysis of the JRM recommendations was conducted for eight countries. An in-depth cross-sectional retrospective analysis of the degree of implementation of these recommendations in Ecuador, Venezuela, Bolivia, and El Salvador was performed through a standardized self-administered questionnaire applied to key informants. A comparative quantitative analysis on the optimization of antiretroviral regimens ‘before/after’ JRMs was conducted in three of the latter four countries, using data reported in 2013 and 2014.

**Results:**

The priority areas with most recommendations were the optimization of antiretroviral treatment (ART) regimens (*n* = 57), the rational and efficient use of resources (*n* = 27) and the provision of point-of-care diagnostics and monitoring tools (*n* = 26), followed by community mobilization (*n* = 23), strategic information (*n* = 17) and the adaptation of delivery services (*n* = 15). The in-depth analysis in four countries showed that the two priority areas where most progress was observed were the rational and efficient use of resources (62 %) and the optimization of ART regimens (60 %). Adaptation of delivery services, community mobilization and strategic information were rated at 52 % and the provision of point-of-care diagnostics and monitoring tools 38 %. The quantitative analysis on optimization evidenced a 36 % reduction in the number of first-line and second-line ART regimens, a 5.4 % increase in the proportion of patients on WHO-recommended first-line regimens, a 19.4 % increase in the use of the WHO preferred first-line regimen, 51 % increase in the use of WHO-recommended second-line regimens, and a significant reduction in the use of obsolete drugs in first- and second-line regimens (respectively 1 and 9 % of regimens in 2013).

**Conclusions:**

A relatively good level of progress was perceived in the recommendations related to optimization of ART regimens. Challenges remain on the improvement of recommendations related to health system strengthening and the promotion and support aimed at community-based organizations as part of the response to HIV/AIDS in Latin America. The JRMs are a useful mechanism for providing coherent technical support to guide countries in the pursuit of a comprehensive response to HIV/AIDS in the Latin American region.

**Electronic supplementary material:**

The online version of this article (doi:10.1186/s12889-015-2565-9) contains supplementary material, which is available to authorized users.

## Background

By the end of 2013 approximately 35 million people were living with HIV (PLHIV) worldwide. Of these, 19 million were unaware that they were HIV-positive, while approximately 13 million were receiving antiretroviral therapy (ART). Of the estimated 1.6 million PLHIV in Latin America by the end of 2013, 70 % had been diagnosed, 44 % of eligible adults (15+ years) and 64 % of the children aged between 0 and 14 years were on ART (2013 World Health Organization [WHO] eligible criteria). Although ART coverage in this Region is the highest among the world’s low and middle-income countries (45 %), rates vary from country to country ranging from 20–64 % (Bolivia and Barbados respectively) [1, 2] and 35 % of new infections are diagnosed late (<200 CD4 cells/μl) [3].

Treatment 2.0 was introduced in June 2010 as an initiative of the WHO and the United Nations Program on HIV/AIDS (UNAIDS) designed to promote the scale-up and sustainability of HIV treatment by enhancing the efficiency and impact of treatment and care programs in countries with limited resources. The initiative responds to the financial and technical challenges that continue to limit universal access to ART [4].

This initiative is intended to guide countries to reach and sustain universal access to HIV treatment and to maximize the preventive benefits of ART through focused work in five interrelated priority areas: optimization of ART regimens, provision of accessible point-of-care (POC) diagnostics and monitoring tools, rational and efficient use of resources, adaptation of delivery systems and community mobilization. These priority areas are interdependent and form a strategic framework for guiding the implementation of the initiative in countries [5].

The concepts of simplification, standardization, community mobilization, and reducing costs are based on the principles derived from WHO’s public health approach to universal access to ART, as well as on scientific evidence and best practices resulting from the implementation of HIV programs [6]. The Treatment 2.0 initiative fits within the UNAIDS Strategy 2011–2015 and the WHO Global Health Sector Strategy on HIV/AIDS 2011–2015 [7, 8]. The expected long term impact of the implementation of this initiative is reduction of morbidity and mortality, as well as the number of new HIV infections. According to UNAIDS-WHO estimates, ten million deaths could be prevented by 2025 [9]. It was recently demonstrated that the effective use of ART is associated with a 96 % risk reduction of sexual transmission of HIV among serodiscordant heterosexual couples as a result of viral suppression in the infected patient. This reinforces new concepts such as prevention benefits of ART, especially when initiated early after HIV infection [10]. If all individuals needing treatment could initiate ART and maintain an undetectable viral load, a third of new HIV infections could be prevented [5, 11].

Engaging the full participation of PLHIV and affected communities in order to sustain the achievements and increase ART coverage rate in the Latin American region is a priority. This will contribute to achieve the commitments made during the 2011 UN General Assembly High Level Meeting on AIDS as well as the new regional care and treatment targets by 2020: 90 % of PLHIV diagnosed; 90 % of eligible people receiving ART; and 90 % of people on ART achieving viral suppression [12].

In recent years Latin American countries have begun to apply the principles of the Treatment 2.0 initiative in their health care programs. The Treatment 2.0 action framework reflects the need to achieve concrete results through simultaneous improvements in each of the initiative’s priority areas. Effective and strategic collaboration between the broad range of stakeholders in the response to HIV is a necessity. The Pan American Health Organization (PAHO) has been supporting this process through various mechanisms among them, working with institutions such as The Council of Ministers of Health of Central America (COMISCA), The Andean Organism for Health (ORAS), the Pan Caribbean Partnership against HIV/AIDS (PANCAP) and the Horizontal Technical Cooperation Group (GCTH).

With technical guidance from PAHO, actions have been pursued in the Region to roll out the Treatment 2.0 initiative. This has been done through various regional consultations and consensus meetings on topics related to ARV stock-outs, HIV testing and counseling, expansion and sustainability of HIV treatment, strategic information as well as the launch of the WHO 2013 consolidated guidelines on ARV drugs for Treatment and Prevention of HIV infection, the WHO 2015 Consolidated Guidelines on HIV testing services 2015 and two Latin American Forums on HIV Care. A series of reports entitled “ART in the Spotlight: a public health analysis in Latin America and the Caribbean” have been published in 2013 and 2014 [3, 13]. In addition, a total of 14 Treatment 2.0 Joint Review Missions (JRMs) had been undertaken up till October 2014: Ecuador (March 2012); Venezuela (May 2012); Bolivia (July 2012); El Salvador (January 2013); the Dominican Republic (February 2013); Honduras (March 2013); Argentina (July 2013); Guatemala (October 2013); Uruguay (November 2013); Nicaragua (March 2014); Paraguay (March 2014); Panama (March 2014); Jamaica (September 2014); and Costa Rica (October 2014).

### Phases of the Joint Review Missions

The typical Treatment 2.0 JRMs format includes three-phases: 1) a preparatory phase during which the team in the country collects and analyzes data on the HIV situation in general and on ART in particular. To ensure broad inclusiveness, an opportunity is provided for in-depth dialogue between national authorities and representatives of civil society and scientific communities; 2) the visits of an international team interacting with all the key actors identified, to finalize the analysis of the situation with special focus on the initiative’s five main priority areas. Based on this situation analysis, visits are prioritized to governmental and nongovernmental facilities providing HIV care and treatment such as hospitals, public health laboratories, pharmacies, national institutes of health, and interviews are held with key informants. At the end of the visit, the joint team develops recommendations and a short and medium-term national work plan for their implementation. The set of the country visit conclusions are presented to the highest representatives of the Ministry of Health and the National AIDS Program to ensure top-level support for implementing the plan; 3) implementation and monitoring of the recommendations.

This study aims to examine the degree of implementation of the Treatment 2.0 initiative in Latin American countries by analyzing to what extent the recommendations resulting from the JRMs have progressed, and assess the impact of these recommendations on health policies.

## Methods

### Selection of countries

In order to assess the degree of implementation of the recommendations, an overall descriptive analysis was conducted among the first eight countries where PAHO and partners undertook this type of review mission: Ecuador, Venezuela, Bolivia, El Salvador, the Dominican Republic, Honduras, Argentina, and Guatemala. An in-depth cross-sectional analysis was also done in four selected countries based on the following criteria: 1) a mission conducted between 12 and 24 months prior to this monitoring exercise; 2) willingness of the national health authorities to participate in this exercise, and; 3) availability of different actors selected for the monitoring exercise. A standardized questionnaire was designed and used among one of each of the following key informants from each country: representative of the national ART programs, representative of civil society linked to ART programs in each country and a technical staff from the PAHO/WHO representation office of each country. The selected countries where this assessment was undertaken were Ecuador, Venezuela, Bolivia, and El Salvador.

### Standardized instrument and the measure of the perception of progress of each recommendation

The standardized tool was designed to collect the information related to the five priority areas of the Treatment 2.0 initiative [see Additional files 1 and 2]. The ‘strategic information’ area was added due to its cross-cutting importance in monitoring the implementation of the recommendations in each country. A color-coded ‘traffic light’ measure was introduced to indicate the progress made in each area, with red representing ‘implementation not yet underway’ , yellow ‘in progress’ and green ‘completed’. In addition, the measurement of the perception of progress of each recommendation was scored on a numerical scale based on the Likert scale, adapted to include different variables ranging from 0–5, where 0 = no progress (0 %), 1 = very early stage (20 %), 2 = initiated (40 %), 3 = in progress (60 %), 4 = at final stage (80 %), and 5 = completed (100 %) [14]. A column was also provided for comments on the implementation of each recommendation. The instrument was piloted by two local technical officers of PAHO in countries were the instrument was not applied to evaluate comprehension and consistency. Results were used to finalize the design of the tool. This standardized instrument was sent to all the selected key informants for self-completion.

A rating scale was established to measure the perception of the interviewees regarding the progress in implementing the recommendations of each priority area. For this, a differential percentage weight for each recommendation was applied to all priority areas, based on established multi-criteria analytical decision-making methodologies [15–17]. Two major factors were also taken into consideration which affected the final attributed weight, the size of the population to be covered by the activity (national, regional or local), and the level of sustainability that the recommendation would achieve in the country. After this first classification was completed, the following secondary factors were applied in order of priority according to attributed percentage weight: a) an activity that needed to be undertaken prior to the other activities, and critical to allow continuity; b) an activity of strategic importance for formulating a national response and in line with international parameters; c) an intermediate activity that could have national impact or seriously interfere in the national process in the specific priority area; d) an intermediate activity with no significant impact on the national response or one that would not negatively impact the other activities in the same priority area; e) a minor activity that could impact on the national response and/or on the implementation of other activities; f) a minor activity with negligible impact on the other activities of the thematic area. The percentage of compliance was calculated based on the average score of recommendations for each priority areas by each key informant from each organization multiplied by the assigned percentage weight for each recommendation. Review of responses and all calculations were done by two of the authors separately (FP, BG) to assure compliance and validity. If different appreciations were found, response results were reviewed and compared and a final result was agreed upon. Incoherent answers were not considered after mutual agreement by the two authors. Results of the percentage of compliance are shown based on the number and type of recommendations by priority areas and country. When a priority area and/or a key informant are not shown, this is because the specific priority area did not have recommendation or the key informant did not respond to the survey.

### Quantitative analysis of the optimization of antiretroviral drugs use

To complement the assessment, a quantitative analysis of the optimization of antiretroviral drugs was conducted in three of the four countries where the implementation of the recommendations was systemically monitored (Bolivia, El Salvador, and Venezuela). This analysis was based on official data reported annually by the countries in the WHO AIDS Medicines and Diagnostics Service questionnaires regarding the use of antiretroviral drugs for 2013 (referring to 2012) and 2014 (referring to 2013) [3, 13, 18]. It was not possible to include Ecuador in this analysis since the country did not report in 2014. The aim was to document changes in the use of antiretrovirals in the countries that had received the Treatment 2.0 JRM, by conducting a comparative data analysis of antiretroviral use in 2012 (before the mission) and 2013 (after the mission). The following indicators were considered in order to compare the average values of the three countries in the two selected years: the number of first-line ART regimens in use; the percentage of patients in first-line ART regimens recommended by WHO (preferred and alternate); the percentage of patients in the preferred first-line ART regimens recommended by WHO (TDF + 3TC [or FTC] + EFV); the percentage of patients in first-line ART regimens including obsolete antiretrovirals (ddl, d4T, IDV, NFV); the number of second-line ART regimens in use; the percentage of patients in second-line ART regimens recommended by WHO; and the percentage of patients in second-line ART regimens including obsolete ARVs.

We reviewed the need for ethics approval with the Pan American Health Organization Ethics Committee who confirmed that given the nature of the study (retrospective survey through interviews) this work did not require ethics approval. All participants were informed about the study and provided oral informed consent to be included. Privacy and confidentiality were maintained throughout the study period; each questionnaire was number-coded without any personal identification.

## Results

### Type and total number of recommendations per country

Ecuador and Bolivia received the highest number of recommendations (29 each), followed by Honduras (*n* = 28) and Argentina (*n* = 20). The technical priority areas where the largest number of recommendations were focused were those corresponding to optimization of ART regimens (*n* = 57), followed by rational and efficient use of resources (*n* = 27) and provision of POC diagnostics and monitoring tools (*n* = 26) (Table 1). The review of the type of recommendations most frequently included in each technical priority area revealed the trends here below:

**Table 1:** Treatment 2.0 Joint Technical Missions in Latin America. Type and number of recommendations by country (2012–2013)

Technical areas	Ecuador	Venezuela	Bolivia	El Salvador	Dominican Republic	Honduras	Argentina	Guatemala	Total
Optimize ART Regimens	10	6	8	10	4	6	5	8	57
Provide point-of-care diagnosis	6	3	4	0	1	8	3	1	26
Reduce costs: rational-efficient use of resources	3	4	4	2	5	5	2	2	27
Adapt delivery systems	0	1	8	0	1	1	4	0	15
Mobilize communities	5	3	1	0	0	6	5	3	23
Strategic information	5	3	4	0	2	1	1	1	17
TOTAL	29	19	29	12	13	28	20	15	165

#### Optimization of ART Regimens:

the following recommendations were present in the eight countries where the monitoring was conducted: 1) to promote the use of regimens of simplified, less toxic drugs that maintain therapeutic efficacy, thus pointing to the need of updating the recommended therapeutic regimens with preferred first-line treatment with TDF + 3TC (or FTC) + EFV in a fixed dosage combination (FDC) for adults and adolescents; 2) the recommended second-line treatment should include two nucleoside reverse transcriptase inhibitors plus a protease inhibitor reinforced with ritonavir (AZT/3TC [CDF]) + ATV/r (or LPV/r) and; 3) the need to gradually phase out ARVs due to their toxicity profile (e.g. stavudine [d4T]) with a migration plan accompanied by an information and communication strategy. In seven of the eight countries, updating the national ART and treatment guidelines (or finalizing this process) was recommended.

#### Provision of Access to POC Diagnostics and Monitoring Tools

four of the eight countries received the following recommendations: 1) the need to develop and implement HIV diagnosis strategies to include POC technologies that would make it possible to expand HIV testing and counseling, mainly at the primary and community level; 2) the use and decentralization of the viral load test as the preferred method for confirming treatment effectiveness and for diagnosis of treatment failure; 3) to decentralize the use of CD4 as a method for evaluating eligibility for ART and for patient monitoring.

#### Rational and efficient use of resources

in seven of the eight countries the following steps were recommended: 1) to adapt the drug and supply management plans considering the services available under the PAHO Strategic Fund, which is a mechanism that facilitates the procurement of strategic public health supplies and materials to the Member States of the Region of the Americas; and 2) to review the methodology for estimating country needs of drugs, supplies and laboratory tests.

#### Adapt Delivery Systems:

the key recommendation in this priority area was related to the need to deconcentrate, decentralize and integrate the ART program at all levels of the healthcare system in four of the eight countries visited.

#### Community Mobilization:

two recommendations were highlighted in this topic: 1) in five of the eight countries there is a need to design and implement communication strategies in order to provide information about the Treatment 2.0 initiative and its priority areas in different national contexts; 2) in three of eight countries it was recommended to involve and engage key populations and people living with HIV in the planning, delivery and evaluation of HIV treatment and care programs. This includes inter alia prevention interventions and support for ART adherence.

#### Strategic Information:

an identified need was to strengthen the information systems in each country in order to have a complete overview of the national cohort of patients in care and treatment including the treatment cascade and plan appropriate and timely interventions. In some countries it is necessary to evaluate and make necessary changes in the existing HIV information systems (MANGUA in Guatemala, CENAVAFELS in Venezuela, and SEGAMI in the Dominican Republic).

### Results of the structured interviews

Overall, representatives from national ART program and international cooperation from each of the four countries completed the questionnaire. Civil society representatives from Ecuador and El Salvador who were identified and agreed to respond to the survey did not submit the completed questionnaire even after various reminders. The reason for this was not established. No incoherent answers were found.

### Analysis of the implementation of the Treatment 2.0 initiative in each country

#### Ecuador

**Fig. 1:**
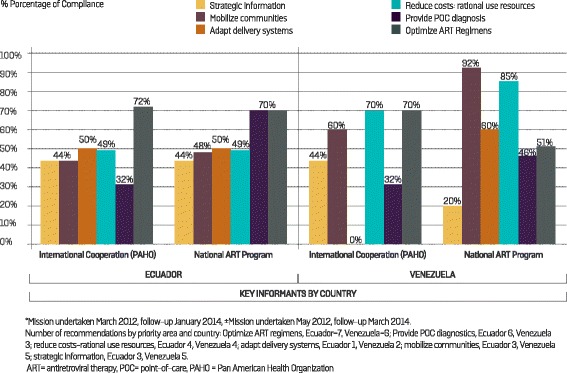
Follow-up of the Treatment 2.0 Strategy in Ecuador (29 recommendations)* and Venezuela (20 recommendations)^±^: perception of the compliance of recommendations by key informants

**Fig. 2:**
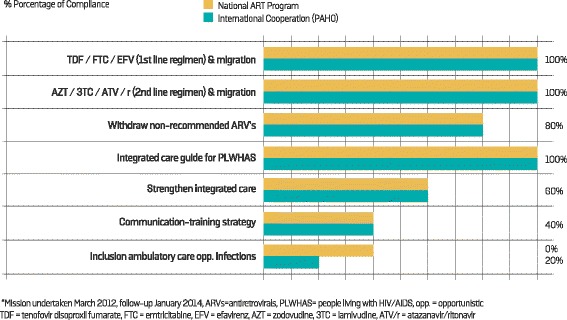
Perception of the compliance of the recommendations regarding the optimization of antiretroviral regimens by representatives of the National AIDS Programof Ecuador and the country PAHO office*

Representatives of the National AIDS Program (NAP) and PAHO responded to the survey for Ecuador. The perception of compliance with the recommendations for each priority area of the Treatment 2.0 initiative in Ecuador shows that most progress was made on the optimization of ART regimens (70–72 %), while the least progress was made on the provision of implementing access to POC diagnostics and monitoring tools (32 %). With the exception of the optimization of ART regimens priority area, none of the other priority areas exceeded 50 % in terms of perception of their implementation (Fig. 1). When examining the optimization of ART regimens priority area, the main recommendation concerned updating the ARV treatment guidelines, together with updating the first and second-line standardized regimens and withdrawing non-recommended drugs (Fig. 2). Finally, both the NAP and PAHO representatives agreed with the perception that little progress had been made in the POC diagnosis priority area implementation (20–40 %) (data not presented).

#### Venezuela

A representative of the NAP and international cooperation representatives for Venezuela (PAHO and UNAIDS) responded to the survey, while it was not possible to gauge the opinion of civil society in order to complete the exercise. The PNS considered that most progress had been made in the community mobilization priority area (92 %), followed by the rational and efficient use of resources priority area (85 %). Meanwhile, representatives from the international cooperation agreed that much progress was evident in the rational and efficient use of resources and optimization of ART regimens (both 70 %), followed by community mobilization (60 %). A significant difference between the respondents was observed in the perception of progress in the adaption of delivery service priority area: the NAP perceived a progress of 60 %, while international cooperation representatives cited that progress was zero. (Fig. 1). Both key informants interviewed agreed that progress on implementation of the recommendations on strategic information was low (20 % NAP, 40 % international cooperation). The strategic information recommendations were perceived by the interviewees to have made limited progress (data not presented).

#### Bolivia

**Fig. 3:**
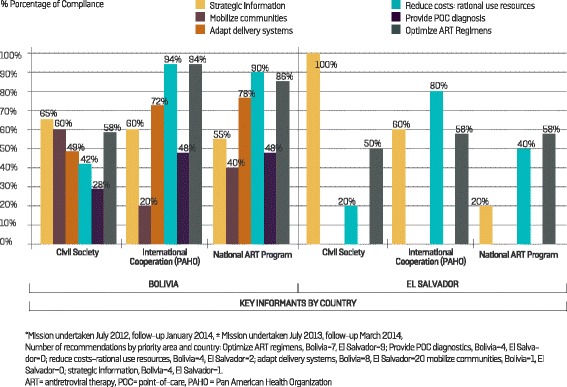
Follow-up of the Treatment 2.0 Strategy in Bolivia (28 recommendations)* and El Salvador (12 recommendations)^±^: perception of the compliance of recommendations by key informants

The monitoring survey for Bolivia was completed by representatives of the NAP, PAHO and civil society. Fig. 3 illustrates the perception of the progress made by the Treatment 2.0 initiative in this country under each priority area. Both NAP and PAHO had a general perception of greater progress than was perceived by civil society. Progress in the optimization of ART regimens priority area scored an average of 79 %, while rational and efficient use of resources was rated at 75 %. The lowest average scores were in the provision of POC diagnostics and monitoring tools (41 %) and community mobilization (40 %) priority areas.

#### El Salvador

Representatives of the National AIDS Program, PAHO and civil society responded to the survey for El Salvador. There were no recommendations for the provision of POC diagnostics and monitoring tools priority area, nor for community mobilization. The area with most recommendations was for the optimization of ART regimens (*n* = 9), where all interviewees concurred that progress had been made (average 55 %). On the other hand, there was a notable difference between the interviewees’ perception of progress in the two priority areas rational and efficient use of resources and strategic information (ranging from 20–100 %) (Fig. 3).

### Comparative analysis of the implementation of each priority area in the different countries

In the four countries where structured interviews were conducted, most perceived progress was made in the rational and efficient use of resources priority area where average progress was perceived to be 62 %, followed by the optimization of ART regimens priority area (average 59.7 %). Meanwhile, progress in the priority areas of the adaption of delivery systems, community mobilization, and strategic information were rated at 52 %. Finally, the provision of POC diagnostics and monitoring tools scored the lowest (an average of only 38 %).

**Fig. 4:**
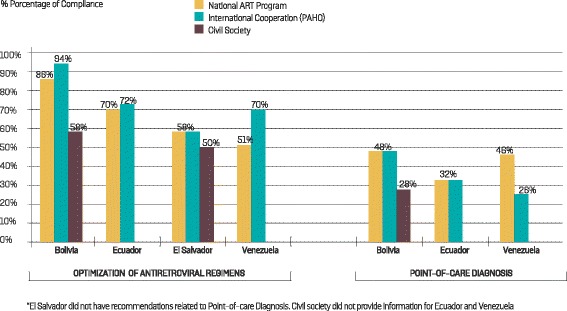
Follow-up of the Treatment 2.0 Strategy in four countries of Latin America. Comparative perception of the compliance of recommendations related to optimization of antiretroviral regimens and provisions of point-of-care diagnostics*

The optimization of ART regimens priority area received an overall average rating of 60 % progress in the four countries (Fig. 4). Detail review of this area showed scores for the recommendation on updating ART guidelines with an 80–100 % progresses in three of the four countries (El Salvador was rated at 60 %). The least progress perceived was related to the recommendation on plans of communication related to the optimization of ART regimens (0–60 %) and ART migration plans (30–60 %) (data not presented).

Limited progress was perceived in the priority area of provision of POC diagnostic and monitoring tools (less than 50 % in all the countries; average = 38 %) (Fig. 4). Although the updating of diagnostic algorithms, with the inclusion of HIV rapid tests, was the most common recommendation, it was perceived to have progressed by only 46 % on average. Meanwhile, the recommendations with the lowest levels of perceived progress were the decentralization of monitoring tests (viral load and CD4), averaging 20 %, and the inclusion of sample transportation strategies, with a perceived 22 % average of implementation (data not included in the graphic).

**Fig. 5:**
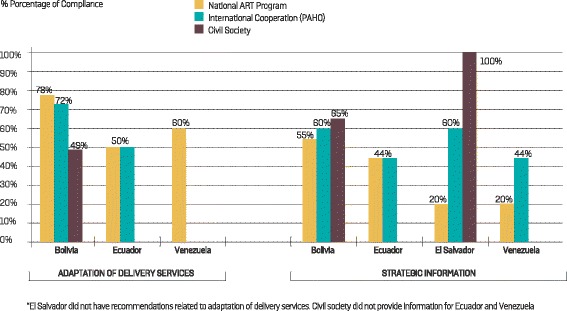
Follow-up of the Treatment 2.0 Strategy in four countries of Latin America. Comparative perception of the compliance of recommendations related to adaptation of delivery services and strategic information*

No recommendations were made on the ‘adaptation of delivery services’ priority area for El Salvador. Of the three remaining countries, Bolivia reported the highest average progress in this area (average 66 % based on responses from all key informants), although there was a substantial gap between interviewees (72 and 78 % for PAHO and NAP respectively, compared to 49 % for civil society). Venezuela had the lowest average perceived progress in this priority area (30 %), although civil society did not respond to the survey. Note that the Venezuelan NAP representatives perceived 60 % progress, while the international cooperation perceived no progress at all. (Fig. 5). The most frequent recommendation in this priority area was the decentralization of health care programs for which the perceived average implementation was scored at 50 % although this was reported in only two countries (Bolivia and Ecuador). The number of recommendations in this priority area was different in each of the four countries: seven recommendations were made for Bolivia, two for Ecuador, one for Venezuela and none for El Salvador.

Recommendations on community mobilization were made in only three countries. Progress achieved in this priority area was on average 50 %. However, it was not possible to obtain a realistic view of the implementation of this priority area in Venezuela and Ecuador since civil society (the main beneficiaries) did not participate in the survey, thereby limiting the value of the analysis. Despite this, Venezuela was perceived to have made progress in terms of community participation (76 %), followed by Ecuador (46 %) and Bolivia (40 %). The most frequent recommendation in this priority area was the need to implement communication and training strategies for civil society, designed to improve understanding and monitoring of the implementation of recommendations related to the other priority areas. The average progress of implementation was perceived at 54 % (data not presented). Implementation of the strategic information recommendations averaged 50 %. El Salvador and Bolivia had the highest perception of progress in this priority area (both with an average of 60 %) (Fig. 5). The most common recommendations in this area were the inclusion and updating of the HIV information system in the general health information systems. The implementation of both recommendations was perceived to have progressed by an average of 30 %.

### Quantitative analysis of the optimization of ARV use

A summary of the results of the analysis of the optimization of antiretroviral use in El Salvador, Venezuela and Bolivia is presented in Table 2. The quantitative analysis shows that there was a 36 % reduction in the average number of first-line ART regimens in use, from 11 in 2012 to seven in 2013. A similar trend can be observed for the second-line ART regimens. Over the same period, the proportion of patients on first-line regimens recommended by WHO increased from 74–78 % (5.4 % increase). The average use of the preferred WHO first-line ART regimen [TDF + 3TC (or FTC) + EFV] increased from 36–43 % (19.4 % increase) between 2012 and 2013 (it should be noted that for this indicator, results are discordant among countries and in Venezuela the use of the WHO preferred regimen declined from 63–43 % over the same time period). In addition, average use of the WHO preferred second-line ART regimens [AZT + 3TC (or TDF + XTC) + LPV/r (or ATV/r)] increased from 43–65 % (51 % increase) between 2012 and 2013. It is also noteworthy that there was a reduction in the use of obsolete ARV in first-line regimens. In 2013, only 1 % of people receiving first-line ART were still on regimens with didanosine (ddl) (no d4T-based regimen was reported). Finally, a reduction in the use of obsolete ARVs in second-line regimens was reported during the period under study. In 2013, 9 % of individuals on second-line ARV were still on regimens with ddl (no d4T-based regimen was reported).

**Table 2:** Optimization of ARVs in the three countries of Latin America, 2012-2013

INDICATOR	Year	El Salvador	Venezuela	Bolivia	Mean
Number of first-line ART regimens in use	2012	11	14	9	11
	2013	12	4	6	7
Percentage of patients in the preferred first-line ART regimens recommended by WHO (TDF + 3TC (or FTC) + EFV)	2012	85 %	39 %	98 %	74 %
	2013	92 %	43 %	99 %	78 %
Percentage of patients in the preferred first-line ART regimens recommended by WHO (TDF + 3TC [or FTC] + EFV)	2012	9 %	63 %	37 %	36 %
	2013	20 %	43 %	66 %	43 %
Percentage of patients in first-line ART regimens including obsolete antiretrovirals (ddl, d4T, IDV, NFV)	2012	13 %	0 %	1 %	5 %
	2013	2 %	0 %	0 %	1 %
Number of second-line ART regimens in use	2012	8 %	18 %	8 %	11 %
	2013	10 %	4 %	7 %	7 %
Percentage of patients in second-line ART regimens recommended by WHO	2012	55 %	0 %	74 %	43 %
	2013	64 %	50 %	83 %	65 %
Percentage of patients in second-line ART regimens including obsolete ARVs	2012	35 %	1 %	19 %	18 %
	2013	20 %	0 %	8 %	9 %

*TDF* tenofovir dispoproxil fumarate, *3TC* lamivudine, *FTC* emtricitabine, *EFV* efavirenz, *ddl* didanosine, *d4T* Stavudine, *IDV* indinavir, *NFV* nelfinavir, *WHO* World Health Organization, *ARVs* antiretrovirals

## Discussion

The priority area with the highest number of recommendations was the optimization of ART regimen, followed by ‘the rational and efficient use of resources’ and provision of POC diagnostics and monitoring tools. In the optimization of ART regimens, seven of the eight countries were recommended to initiate or finalize the process of updating their national ART guidelines in accordance with the WHO 2013 recommendations. This implies, promoting the use of simplified, less toxic regimens that maintain therapeutic efficacy, as well as accelerating the gradual elimination of obsolete ARV drugs that are no longer recommended. Four of the eight countries received recommendations on POC diagnosis and monitoring, underscoring the need to develop and implement HIV diagnostic strategies to include POC technologies and the decentralization of viral load and CD4 testing. In the four countries where detailed monitoring of the recommendations was conducted, the highest proportion of the recommendations was related to the optimization of ART regimens, and a good level of progress was perceived in several of the recommendations related to this area (59.7 % average). This could be explained by the fact that the countries already had previous experience in the process of periodic update of their national ARV guidelines and in some countries the process was already undergoing.

The three countries evaluated on the basis of the reported quantitative data show signs of improvement in the more strategic use of antiretroviral regimens and drugs. There has been a marked reduction in the number of first-and second-line regimens, a greater adherence to the WHO recommendations, especially regarding the increased use of the preferred first-line regimen, and the almost total withdrawal of obsolete drugs from the HIV therapeutic arsenal [19]. These same trends have been observed in many countries of the Latin American and Caribbean region [13].

With regard to the rational and efficient utilization of resources, ARVs remain one of the most expensive components, representing a significant part of the health budgets of low- and middle-income countries of the Latin American and Caribbean region. It is important to ensure the efficient procurement of these drugs. Recent studies reveal the existence of significant price variations for ARVs among the countries of the region and opportunities for cost savings in the procurement of these essential drugs need to be explored [20]. Efficient resource utilization signals the need for an integrated approach to purchasing and delivering ARV. The PAHO Strategic Fund procurement process is one approach [19].

It is necessary to emphasize that the key informants of the four countries reported slow progress in the areas requiring high-level decision-making and investment of major resources for strengthening the health system or enhancing multi-sectorial coordination and/or integration. One example of this is the need to develop and implement HIV diagnosis strategies that include POC technologies and the use and decentralization of viral load and CD4 testing (average 38 % perceived progress in the POC diagnosis and monitoring priority area). Based on the results of recent evaluations, the increased use of virologic monitoring as the preferred monitoring approach is foreseen in the near future [21, 22]. Operational actions related to deconcentration, decentralization, and the integration of ART programs at all levels of the healthcare system have been more difficult to implement, probably due to the need to involve various multi-sectorial health areas in a country beyond the National AIDS Programs. For Latin American countries to adopt the 2013 WHO consolidated HIV treatment and prevention guidelines, they will have to invest in the diversification of health service delivery models in order to cope with increased volumes of patients and ensure quality of care in the cascade of care and treatment [23].

When formulating HIV care and treatment policies, community mobilization approaches are important for ensuring that the interventions respond to the needs and circumstances of people at greatest risk and those living with HIV [24]. The present evaluation concluded that the average perceived level of progress in the two countries (where information was available) was near 50 %, allowing for variations from country to country. The traditional role of civil society has been critical to the response of the HIV/AIDS epidemic. It is necessary to continue to promote and support community mobilization in order to ensure the sustainability of the response to HIV/AIDS in the region and assure the involvement of representatives of civil society in country HIV/AIDS program assessments.

Finally, although it is not possible to attribute country progress of the implementation of the priority areas of the Treatment 2.0 initiative directly and solely to the activities of the JRMs, the review of how country recommendations have been applied suggests that the JRMs have played a key role by supporting the countries to create a solid basis for a coordinated response. This is leading to more rapid expansion of ART coverage and considering quality of care. In most cases, the country visits have had a catalyzing effect given that they were able to bring together key actors at national level and provide support to reach consensus on national ART policies.

Potential limitations to the methodology used for monitoring the recommendations arising from the JRMs must be acknowledged. Approximately 15 months have elapsed between the country visits and the present review. We recognize that it is not feasible to accurately assess the progress achieved in each country concerning the different Treatment 2.0 priority areas only on the basis of interviews with key informants. In addition, the use of a self-reported questionnaire could have allowed bias in the process of data recording. A third consideration relates to the potential limitations in the progress rating scales which are dependent on a number of different factors such as the local context and the structure of the questionnaire [25, 26]. In order to mitigate these limitations, we monitored the recommendations made by the JRMs by using key informants with knowledge of a particular community or of a relevant problem area. To avoid professional [27] and recall bias [28], the information provided by key informants was validated through interviews with several different stakeholders: a representative of the National HIV/AIDS Program, a civil society representative and an international cooperation representative (PAHO country office). We realize however that this approach cannot fully ascertain the degree to which the specific recommendations of the JRMs have been implemented in a particular country. Therefore we consider the need to undertake periodic monitoring visits, using specific and measurable verification indicators, in order to objectively assess progress in the implementation of the recommendations. This should involve developing national plans and improving access to additional resources such as the Global Fund to Fight AIDS, Tuberculosis and Malaria [29].

## Conclusions

A relatively good level of progress was perceived in the recommendations related to optimization of ART regimens. Challenges remain on the improvement of recommendations related to health system strengthening and the promotion and support aimed at community-based organizations as part of the response to HIV/AIDs in Latin America. Although there is still a need to increase the impact of the JRMs in the countries, the monitoring exercise confirms that these missions are a useful mechanism for providing coherent technical support to guide countries towards formulating a comprehensive response to HIV/AIDS in the Latin American region. The results also suggest that periodic follow-up of the recommendations of the Treatment 2.0 JRMs is useful and necessary in order to boost their implementation and detect areas requiring more programmatic attention and technical assistance.

## Additional files

Additional file 1:
**Questionnaire Spanish version.** (XLSX 61 kb) Additional file 2:
**Questionnaire English version.** (XLSX 48 kb)

## Abbreviations

3 T: Lamivudine
AIDS: acquired immune deficiency syndrome
ART: antiretroviral therapy
ATV/r: Atazanavir/ritonavir
AZT: Zidovudine
COMISCA: The Council of Ministers of Health of Central America
ddl: Didanosine
ddl: Stavudine
EFV: Emtricitabine Efavirenz
FDC: fixed dosage combination
FTC: Emtricitabine
GCTH: The Horizontal Technical Cooperation Group
IDV: Indinavir
JRMs: Joint Review Missions
LPV/r: Lopinavir/ritonavir
NAP: National AIDS Program
NFV: Nelfinavir
ORAS: The Andean Organism for Health
PAHO: Pan American Health Organization
PANCAP: The Pan Caribbean Partnership against HIV/AIDS
POC: point of care
TDF: Tenofovir dispoproxil fumarate
UNAIDS: United National AIDS
WHO: World Health Organization
